# Evaluation of Therapeutic Tissue Crosslinking (TXL) for Myopia Using Second Harmonic Generation Signal Microscopy in Rabbit Sclera

**DOI:** 10.1167/iovs.16-20241

**Published:** 2017-01

**Authors:** Mariya Zyablitskaya, Anna Takaoka, Emilia L. Munteanu, Takayuki Nagasaki, Stephen L. Trokel, David C. Paik

**Affiliations:** 1 Department of Ophthalmology, Columbia University College of Physicians and Surgeons, New York, New York, United States; 2 Confocal and Specialized Microscopy Shared Resource, Herbert Irving Comprehensive Cancer Center, Columbia University, New York, New York, United States

**Keywords:** sodium hydroxymethylglycinate, tissue crosslinking, high myopia, sclera, second harmonic generation microscopy

## Abstract

**Purpose:**

Second harmonic generation signals (SHG) are emitted preferentially from collagenous tissue structures and have been used to evaluate photochemically-induced (CXL) crosslinking changes in the cornea. Since therapeutic tissue crosslinking (TXL) using sodium hydroxymethylglycinate (SMG) of the sclera is a potential treatment for high myopia, we explored the use of SHG microscopy to evaluate the effects.

**Methods:**

Single sub-Tenon's (sT) injections (400 μL) using SMG (40–400 mM) were made at the equatorial 12 o'clock position of the right eye of cadaveric rabbit heads (*n* = 16 pairs). After 3.5 hours, confocal microscopy (CM) was performed using 860 nm two-photon excitation and 400 to 450 nm emission. Pixel density and fiber bundle “waviness” analyses were performed on the images. Crosslinking effects were confirmed using thermal denaturation (Tm) temperature. Comparison experiments with riboflavin photochemical crosslinking were done.

**Results:**

Therapeutic tissue crosslinking localization studies indicated that crosslinking changes occurred at the site of injection and in adjacent sectors. Second harmonic generation signals revealed large fibrous collagenous bundled structures that displayed various degrees of waviness. Histogram analysis showed a nearly 6-fold signal increase in 400 mM SMG over 40 mM. This corresponded to a ΔTm = 13°C for 400 mM versus ΔTm = 4°C for 40 mM. Waviness analysis indicated increased fiber straightening as a result of SMG CXL.

**Conclusions:**

Second harmonic generation signal intensity and fiber bundle waviness is altered by scleral tissue crosslinking using SMG. These changes provide insights into the macromolecular changes that are induced by therapeutic crosslinking technology and may provide a method to evaluate connective tissue protein changes induced by scleral crosslinking therapies.

Progressive myopia currently is postulated to be potentially treatable through scleral crosslinking, which makes sense given that blocking collagen crosslinking can increase form-deprivation (FD)–induced myopia.^1^ Elsheikh and Phillips^2^ recently have discussed the feasibility and potential of using riboflavin photochemistry (as has been used on the cornea for the treatment of keratoconus), for posterior scleral stabilization to halt axial elongation. Although successfully used for treating destabilization of the anterior globe surface, that is, the corneal bulging seen in keratoconus, accessing the opposite end of the globe for posterior scleral crosslinking requires surgical access to the region, which has its own set of associated issues. Previous attempts at scleral crosslinking (sCXL) using UVA-riboflavin–mediated photochemical crosslinking (CXL) also were reported, although difficulty accessing the posterior sclera with an ultraviolet (UV) light source was a concern^3,4^ as well as changes in electroretinographic (ERG) amplitudes following CXL in rabbit sclera.^5^ More recently, the “CXL” approach has been used successfully to halt axial elongation in visually form-deprived rabbits (by tarsorrhaphy), although multiple regions of posterior sclera required separate irradiation zones.^6^

Although the CXL technique is being used successfully for stabilizing the cornea, treating the sclera in the same way presents a different set of challenges and may not be the best method to induce tissue mechanical property change. In this regard, injection of a chemical stabilizing agent via the sub-Tenon's (sT) space could represent a simpler way to treat the posterior sclera, avoiding the need for UV light exposure. This technique is well known as a useful method of inducing ocular anesthesia.^7–9^ Wollensak^10^ has described previously the use of a sT injection using glyceraldehyde (a chemical crosslinking agent similar in concept to the FARs described in this study) to stiffen the rabbit sclera^10^ and genipin has been shown to limit axial length in FD guinea pigs.^11,12^ These investigators have demonstrated an advantage of using a soluble chemical agent over the photochemical (CXL) technique. Making an injection into sT space is much simpler than carrying out the photochemical procedure, which requires a surgical procedure to access the posterior sclera with a UV light source. Thus, scleral crosslinking using an injectable chemical agent of some type, such as the FARs,^13^ could provide a means to halt the progression of scleral elongation seen in myopia.

Our approach is to use a chemical crosslinking solution of sodium hydroxymethylglycinate (SMG), delivered via sT injection. In previous studies from our lab we have been applying this chemical crosslinking agent to the cornea, but changing the target tissue for crosslinking to sclera could be useful. The previous experiments indicated that a concentration-dependent crosslinking effect could be obtained using SMG, with effects ranging well above those achieved with standard CXL as determined by thermal analysis.^14^

Second harmonic generation microscopy (SHGM) is a promising method for evaluating therapeutic collagen crosslinking. It has been known for more than 30 years that fibrillar collagens in tissues emit SHG signal.^15^ However, only recently could high-resolution images be obtained.^16^ Collagen is uniquely suited for imaging by SHG signal and is the major SHG signal generator in the extracellular space. Second harmonic generation signals using LSM have been used successfully for imaging collagens in a variety of tissues, including tendon,^17^ skin, cartilage,^18^ blood vessels,^19^ and in collagen gels.^20^ Collagen's unique noncentrosymmetric structure is responsible for the large signal generated and much has been written on this subject,^20,21^ including the fact that SHG signal is generated from the fibril surface as a “tube-like” structure.^17^

Previous tissue crosslinking studies using CXL examined by SHG imaging have been reported but similar studies have not been performed for scleral crosslinking to the best of our knowledge. Based on this knowledge, we undertook to study the SHG signal changes induced in the sclera through crosslinking technology, focusing on a crosslinking agent that our lab has been interested in using as a potential way to limit axial elongation in progressive myopia.^13^ The results indicate that chemical crosslinking of the sclera using SMG increases the SHG signals produced from tissue collagens and may induce a structural morphologic change in the collagen fiber network.

## Methods and Materials

### Chemicals

Sodium hydroxymethylglycinate and sodium bicarbonate, were obtained from Tyger Chemicals Scientific, Inc. (Ewing, NJ, USA) and Sigma-Aldrich Corp. (St. Louis, MO, USA) respectively. All chemical solutions and buffers were prepared fresh using Millipore water (double distilled, de-ionized water, *ρ* = 18.2 MΩcm at 25 °C) on the day of crosslinking.

Each solution was prepared within 20 minutes of application. All dilutions were made with distilled H_2_O. The pH measurements of SMG (40–400 mM) and NaHCO_3_ (200 mM) were 10.7 and 8.5, respectively. Sub-Tenon's injected globes were given a single injection of 400 μL volume. The incubation time was 3.5 hours at room temperature (18°–20°C), at which time the tissue was dissected from the globes, washed in PBS three times and left to soak in fresh solution while immediately transporting for microscopy. It is possible that some degree of endogenous tissue degradation may have occurred during this time period. However, we wanted to avoid cold storage conditions to allow the reactions to proceed under simulated physiologic conditions. In other words, we wanted to provide time to allow the compound to fully permeate the tissues as well as allow for the crosslinking reactions to go to completion at a temperature approaching physiologic. Our room temperature of 18° to 20°C is a compromise in this regard (between cold storage and body temperature, that is). In addition, no microscopy findings (as viewed by confocal microscopy) were noted that would suggest significant tissue degradation.

### Eyes

All procedures were performed according to the ARVO Statement on the Use of Animals in Ophthalmic and Vision Research.

### Tissue Crosslinking Procedures

#### Tissue Crosslinking.

We used 16 pairs of eyes for the experiments that were delivered to the lab as intact rabbit heads from a local abattoir within an hour of being euthanized. The right eyes received TXL treatment, whereas the left eyes received a mock injection using 200 mM NaHCO3. The injection site was stained with a marker to facilitate identification for microscopy. The area of injection was the superior nasal quadrant. Intraocular pressure of each eye was measured with a Tono-pen (Reichter Technologies, Depew, NY, USA) before and after injection and found to be similar between globes and close to the normal range for New Zealand White (NZW) rabbits (6–11 mm Hg).^22^

Additional control experiments also indicated that IOP did not change over the course of our 3.5-hour experiment. The sT injection was made using an insulin needle (25 gauge = 0.5 mm). The sT space was identified by passing through the conjunctival and Tenon's capsule layers with the needle. The needle was moved from side to side to confirm that the globe had not been entered inadvertently and to introduce the TXL solution (400 μL volume). Conjunctival swelling was observed routinely following the injection. Following injection, the rabbit head was left at room temperature (18°–20°C) for 3.5 hours, after which the marked injection site was excised in the anteroposterior direction beginning 2 mm from the limbus. In this way, scleral strips were made approximately 5 × 8 mm in size. The retina and choroid was scrubbed off the underside of the sclera during tissue dissection to provide a cleaned sclera tissue strip. The outer surface, including conjunctiva and muscles, then was removed as well. The excised, cleaned scleral tissue piece then was washed in PBS twice and then placed in PBS solution and transferred to the microscopy facility for imaging.

To examine how the effect of treatment spreads from the site of injection, we conducted a series of experiments examining the localization of crosslinking effect induced. To do this, the globes were divided anatomically into 16 sectors, 9 from the top, 2 from each side, and 3 from the bottom. The injection site corresponded to sector 2, a 4 × 4 mm area located just anterior to the equator at the 12 o'clock position. Sectors 1 and 3 were approximately 4 × 4 mm square segments taken adjacent to the injection site extending to 5 mm posterior to the limbus anteriorly, and 1.5 cm anterior to the posterior pole posteriorly. These anterior and posterior “margins” represented the extent of tissue sectioning for the remaining sectors as well (4–16). Sectors 4 to 6 were taken from the adjacent superonasal location and sectors 7 to 9 were taken from the adjacent superotemporal location. Sectors 10 and 11 were taken from the medial orbital wall and sectors 12 and 13 were taken from the lateral orbital wall. Sector 15 was taken from the 6 o'clock location with sectors 14 and 16 taken from the adjacent regions. In this way, we determined the denaturation temperature for each sector independently. This allowed for a general determination of effect localization.

#### UVA-Riboflavin–Mediated Photochemical Crosslinking.

The standard corneal CXL procedure was adopted (with some modification) from the “Dresden protocol”^23^ but applied to scleral crosslinking on a restricted scleral region by rotating the intact eyeball. This was achieved by incising the rectus muscles on the superior and temporal sides, followed by immobilization by suturing the sclera to the lower eyelid. Photochemical corneal crosslinking light source parameters were used: wavelength 370 nm for 30 minutes. The power was 1.5 mW and the intensity was 2.984 mW/cm2, to deliver a dose of 5.371 J/cm2 using the Opto Corneal Crosslinking System (Opto Electronica, Sao Carlos, Brazil). A solution of riboflavin-5-phosphate diluted in 1.1% (hydroxypropyl)methyl cellulose (HPMC; 15 centipoise) was made up in dH2O and applied every 3 minutes throughout the irradiation procedure, which lasted exactly 30 minutes. The distance from the light source to the surface of the sclera was maintained at exactly 45 mm. We did not use high molecular weight dextran for our riboflavin solution (as described in the Dresden protocol) but instead used 1.1% HPMC for our riboflavin solution. The HPMC is in clinical use as a substitute for the dextran used in the original UVA-riboflavin studies and we used HPMC in this study.

### SHGM

Image collection, processing, and analysis were performed at the Confocal and Specialized Microscopy Shared Resource of the Herbert Irving Comprehensive Cancer Center at Columbia University. As a methods control, solutions containing SMG and riboflavin-5-phosphate were measured for SHG signal artifact as well as scleral tissue that had been soaked and then washed free of riboflavin solution (2 × 5 minutes wash followed by soak during transport).

Tissue was placed with the outer sclera facing down on a round coverslip (thickness #1.5, diameter 25 mm; Warner Instruments, Hamden, CT, USA) mounted in a closed metallic chamber well (AttoFluor cell chamber; Molecular Probes, Eugene, OR, USA). For imaging we used a Nikon A1R-MP laser scanning system on an Eclipse Ti microscope stand (Nikon Instruments, Melville, NY, USA) equipped with a Nikon 25x/NA1.1 Apo LWD water-immersion objective. The samples were excited with 860 nm laser light for SHG signals and at 700 nm for autoflourescence imaging from a Chameleon Vision II tunable laser (Coherent, Santa Clara, CA, USA). The back-scattered SHG emission as well as autofluorescence was detected with a nondescanned detector using a 400 to 450 nm bandpass filter. Laser power was 60 mW (2.5% of full power) at 860 nm and the laser pulse width was 140 fs at 80 MHz. Pixel dwell time was 6.2 × 10^–9^ sec/pixel. Resolution of images was 1024 pixels/line. Image capture was done automatically using NIS Elements software (version 4.3; Nikon Instruments Inc., NY, USA).

The area of captured image was chosen by scanning through all tissue with the microscope (previewing) and capturing only the ones where tissue was well visualized over the entire image field and not overlapping. For concentration-dependent comparison studies, the depth was adjusted on each image to be approximately 10 to 15 μm from the episclera. A top coverslip also was used in a “sandwich” method to minimize tissue irregularities, such as folds or wrinkles, that can create a visibly darker area on the image if present at a different depth.

### Image Analysis

Pictures were further analyzed using Fiji software.^24^ Pixel density, as a reflection of signal brightness, was obtained from each image by plotting a histogram and taking its mean value. Differences in mean pixel density values between treated and control samples then were examined. In addition, an analysis of waviness was done using methods adopted from the cardiovascular blood vessel literature, which uses the NeuronJ^25^ plugin for the FIJI software. The end-to-end fiber length was taken using a computer mouse controlled drawing tool with images of single focal planes. The straight line distance from each end of the traced fiber then was measured and expressed as the linear distance. Then, the waviness % was defined as: Waviness-% = (Waviness[SMG] − 1)/(Waviness[control] − 1), where Waviness[ ] = Length[curve]/Length[linear]. For this analysis, a minimum of 10 images per crosslinking condition for each independent determination were taken for analysis. Ten fiber measurements were taken within each image field and 10 image fields were evaluated for each crosslinking condition. For each image, the 10 fiber measurements were first averaged. Then the values for the 10 images were averaged and statistical analysis performed.

### Differential Scanning Calorimetry (DSC)

To confirm the crosslinking effect, all samples underwent thermal denaturation analysis (Perkin Elmer DSC 6000, Waltham, MA, USA). Tissue samples were transferred to preweighed 50-μL aluminum pans. Peak thermal denaturation temperature (Tm) of all 6 mm scleral buttons was measured using a Perkin-Elmer DSC 6000 Autosampler. The difference in thermal denaturation temperature (ΔTm) between the crosslinked sample and contralateral paired control sample was determined using the Pyris software (version 11.0; Perkin-Elmer) as previously reported.^13^

### Statistical Analysis

Using the aforementioned Fiji software, histogram analysis of each image yielded a mean pixel density value. This mean was the outcome value used in the analysis. To compare different doses, we fit a linear mixed model with a random intercept for rabbit. The outcome in the model was the mean from the histograms, and the predictors in the model were the different concentrations of crosslinker. From this model, we estimated the mean differences for all pairwise comparisons of concentrations (i.e., 40 vs. 0, 400 vs. 0). To estimate the localization effect of crosslinking solution, each sector from the treated globe then was compared to the corresponding sector from the control globe (i.e., the contralateral eye from the same rabbit head or paired control eye), using paired *t*-tests. We also report means and standard deviations to describe these differences. Statistical significance of all tests was based on an α value of 0.05 (*P* < 0.05). Calculations were done using STATA Statistical analysis software (StataSE 13; StataCorp, College Station, TX, USA). Significance of Tm, “straightening effect” analysis, and parts of the histogram analysis were calculated using Microsoft Excel (Microsoft Corporation, Redmond, WA, USA).

## Results

### Studies Using Tm as an Assay Method to Evaluate TXL Crosslinking Effect

**Figure 1:**
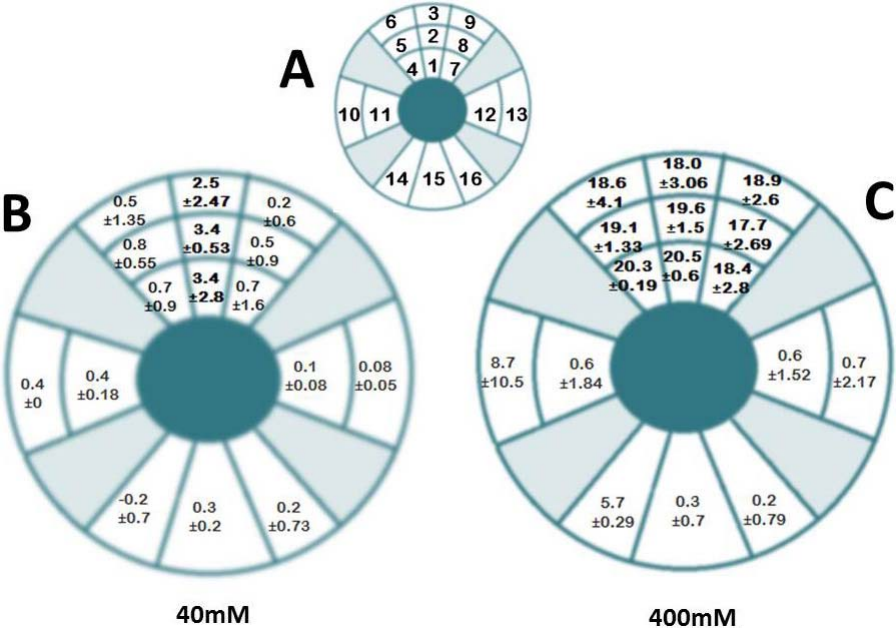
Localization of TXL effect sT injection using 40 and 400 mM SMG. A single injection of SMG solution was applied via the sT space using a 25-gauge insulin needle and syringe. Following a 3.5-hour incubation, the tissue was sampled at a series of locations by sectioning the tissue. Each sector was analyzed independently for crosslinking effect by DSC. The corresponding sectors in the contralateral NaHCO3 injected eyes were treated and sectioned in a similar fashion and were expressed as the difference in Tm, or ΔTm. (**A**) Represents a schematic of the location of injection and excised scleral sectors and is not drawn to scale. The injection site corresponded to sector 2, a 4 × 4 mm area located just anterior to the equator at the 12 o'clock position. Sectors 1 and 3 were approximately 4 × 4 mm square segments taken adjacent to the injection site extending to 5 mm posterior to the limbus anteriorly, and 1.5 cm anterior to the posterior pole posteriorly. These anterior and posterior “margins” represented the extent of tissue sectioning for the remaining sectors as well (4–16). Sectors 4 to 6 were taken from the adjacent superonasal location and sectors 7 to 9 were taken from the adjacent superotemporal location. Sectors 10 and 11 were taken from the medial orbital wall and sectors 12 and 13 were taken from the lateral orbital wall. Sector 15 was taken from the 6 o'clock location with sectors 14 and 16 taken from the adjacent regions. (**B**) 40 mM SMG and (**C**) 400 mM SMG are the localization maps for SMG crosslinking. Each sector contains the ΔTm ± SD, representing the shift in denaturation temperature. That is the mean difference in Tm between experimental sets and their corresponding paired controls. The results indicate that a localization of effect occurs in the region of the injection. In 400 mM–treated samples, the localization of effect is similar to 40 mM–treated samples, but encompasses a greater area with significantly greater crosslinking effects, as evidenced by the large shifts in Tm (18.0°–20.5° vs. 2.5°^–^3.4°C). Each value represents the average of a minimum of 3 independent determinations. *P* values for each sector are not shown but sectors showing statistically significant values were sectors 1 to 3 for 40 mM (**B**) and 1 to 9 for 400 mM (**C**).

As the initial portion of the study, we began by looking at the localization of crosslinking effect induced by injection of our TXL crosslinking agent following sT injection in the cadaveric rabbit head. This type of experiment has relevance to the clinical treatment of patients, since injections in more than one location could be necessary to stabilize a desired area of sclera. As would be predicted based on basic diffusivity principles, the effect was greatest at the site of injection with effects induced in adjacent regions as well, depending on the concentration of the solutions. Figure 1A represents the schematic location of scleral sectors that underwent separate thermal denaturation following a single sT injection with color mapping index. Figures 1B and 1C represent the results using two different concentrations of SMG, 40 mM (Fig. 1B) and 400 mM (Fig. 1C). In Figure 1B, the lower concentration 40 mM sample, a 3.4°C shift in Tm was noted in sector 2 (the injection site), with similar shift seen in adjacent sectors 1 and 3. Marginal shifts are seen in sectors 4 to 6 and 7 to 9 without statistically significant differences and no effect was seen in the lower sectors 14 to 16.

As shown in Figure 1C, the higher concentration (400 mM) had a statistically highly significant crosslinking effect. A large shift in Tm with associated small standard deviation and *P* value were observed, reflecting a large difference in the effect of the 400 mM compared to lower 40 mM concentration. The effects were noted in sectors 1 to 3 as well as 4 to 6 and 5 to 9 in the upper globe. With regard to the remaining sectors, a lesser effect was observed in sectors 10 and 14 (which may have been due to some tracking of crosslinking fluid posteriorly) and no effect in sectors 11, 12, 13, 15, and 16. The effect is marginal in the lateral and medial sectors 10 and 11, and 12 and 13, with no effect in lower sectors 14 to 16, similar to the 40 mM sample. These results indicated that there is a “zone of effect” that should be expected following a sT injection of crosslinking agent, such that adjacent injections could be needed to cover a wider area.

**Figure 2:**
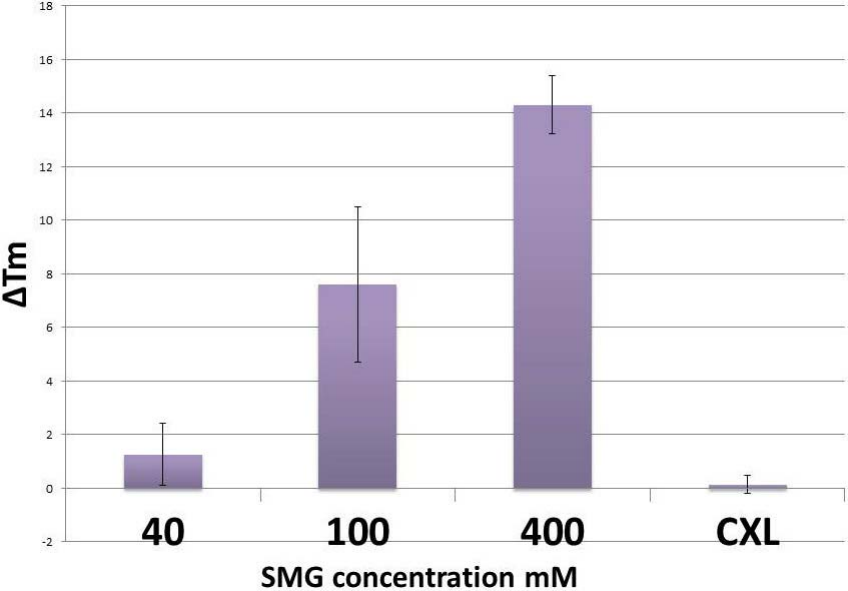
Concentration-dependent thermal denaturation effects using SMG via sT injection and photochemical crosslinking in ex vivo in situ rabbit eyes. Methods as described previously (see text). The difference in TXL effect taken from the site of injection based on results from Figure 1, varies depending on the concentration of SMG used. No crosslinking effect was observed using the “CXL” riboflavin photochemical crosslinking technique.

We next performed a study comparing the concentration-dependent crosslinking effects induced in intact globes compared to riboflavin photochemical crosslinking (CXL). Figure 2 represents the thermal shrinkage analysis of scleral TXL performed in two different ways, first by performing a single sT injection using SMG on intact eyes left in situ, and second, by carrying out riboflavin photochemical crosslinking. Crosslinking time was 3.5 hours and three concentrations, 40, 100, and 400 mM, were used. The results showed that there is a concentration-dependent effect seen in SMG crosslinked tissue. A similar increase in CXL-treated tissue was not observed and the reasons for this could be related to the fact that scleral tissue crosslinking may require a different irradiation protocol, including higher light irradiances. Also, tissue riboflavin penetration of the sclera was not specifically studied and also could have contributed to the lack of effect. In this study, we adopted a modified method from CXL corneal crosslinking (using HPMC in place of high molecular weight dextran) studies (commonly known as the Dresden protocol). In addition, a single area was chosen for crosslinking using the UVA-riboflavin treatment although this should not have impacted the Tm results, since the area samples for Tm analysis was the exact injection site region.

### SHG Imaging

To prove that SMG solution itself did not modulate the SHG signal in any way, a series of SMG solutions were tested for SHG signal generation at various excitation wavelengths (700–860 nm). Negligible levels of SHG signal were generated from SMG in solution alone (data not shown). A similar methods control experiment was performed using a riboflavin-5-phosphate solution to determine if riboflavin caused any augmentation of the SHG signal. Riboflavin is known to be absorbed in the visible light region above 400 nm (380–750 nm). Because the collagen SHG signal is captured from 400 to 450 nm (from an 860-nm near-infrared laser), an overlap region exists between the SHG signal generated from collagen and the absorption spectrum emanating from riboflavin. The experimental results showed that riboflavin, when soaked into the scleral tissue, indeed caused an increase in signal absorption in the 435 to 440 nm region, overlapping with the SHG signal (430 nm). This measurement artifact could be minimized by aggressive rinsing of the tissue in PBS (data not shown).

**Figure 3:**
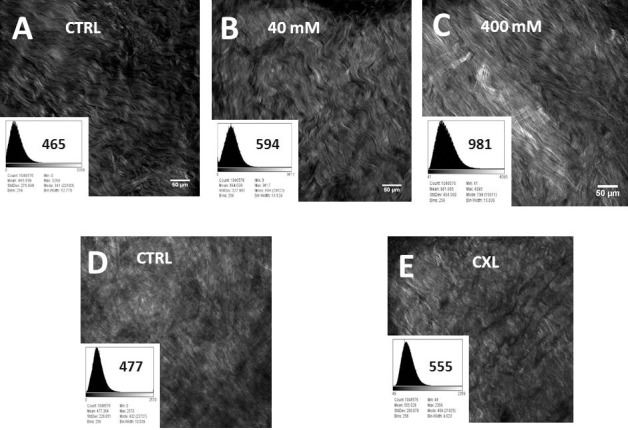
Representative images of concentration dependent increases in SHG signal brightness levels produced following induced crosslinking (TXL) using SMG via sT injection and by UVA-riboflavin crosslinking of sclera ex vivo. Each sample has its own paired control. Two different concentration of SMG are shown as (**B**) 40 mM and (**C**) 400 mM, as well as (**D**) riboflavin only control and (**E**) CXL crosslinking. The absorption spectrum of riboflavin (max ca. 420 nm) is known to overlap with the SHG signal generated (860 nm excitation with 400–450 nm emission) and this could confound the SHG signal although washing the tissue three times in PBS was sufficient to generate baseline signals without artifact (data not shown). The histogram analysis of pixel density accompanies each Figure and is computer software generated (NIS software version 4.3). Statistical comparisons were then performed as shown in Figure 4.

**Figure 4:**
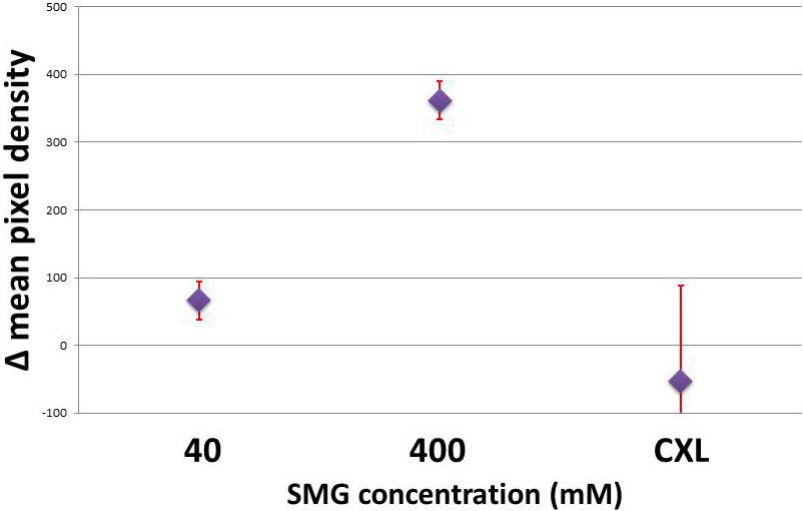
Average of the differences in densitometric SHG signals (mean pixel densities) in scleral intact globes crosslinked via sT injection with 40 and 400 mM SMG solutions, as well as riboflavin photochemical crosslinking (CXL). A nearly 6-fold increase in SHG signal brightness is noted in the 400 mM sample over 40 mM SMG. Photochemically crosslinked samples did not show increased signal as compared to paired control samples.

Images taken with SHG signal confocal microscopy were analyzed by plotting a histogram of each image and taking averages of pixels' density at approximated 1 million different coordinates. We evaluated the SHG signals produced over a range of concentrations spanning from 40 to 400 mM. Our intention was to explore SHG signal changes over a wide range of crosslinking effects. Using the histogram analysis capability included in the NIS Elements software package (version 4.3), we were able to quantitate the SHG signal produced in scleral tissue by sT injection, comparing the effects at 40 mM to those induced using 400 mM. Average difference in mean pixel densities at 40 mM were 66.3 ± 27.7 compared to 361.4 ± 28.3 for the 400 mM samples, a nearly 6-fold increase. This corresponds with an increase in tissue crosslinking, since corresponding increases in Tm also were noted under these conditions. Figure 3 shows representative SHG images of sclera taken from control (Fig. 3A), 40 mM (Fig. 3B), and 400 mM (Fig. 3C) SMG-treated samples by sT injection; riboflavin-only control (Fig. 3D); and (Fig. 3E) riboflavin photochemical crosslinking (CXL). The accompanying histogram analysis, including mean brightness (or pixel density) is shown in the lower corner of each image. The depth of tissue imaging is 10 to 15 μm from the episcleral surface. The results of the histogram analyses, which involved the averaging of numerous image fields, indicated that higher concentrations of crosslinking solution and ultimately higher degrees of crosslinking effect correlate with increased SHG signal (brightness of image). Shown in Figure 4 is the difference in average pixel densities for SMG and CXL-treated samples. Statistical significance (*P* < 0.05) was observed for both concentrations of SMG. The average pixel density for the photochemical crosslinking did not show any increase in signal brightness and was in agreement with the Tm results that showed no shift in thermal stability in the CXL group indicating lack of crosslinking effect (Fig. 2).

**Figure 5:**
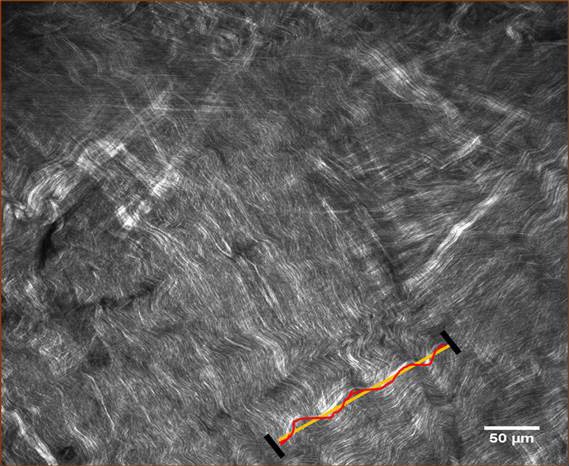
Representative image and example of fiber waviness analysis (as expressed by linearity). Quantitative collagen fiber waviness analysis was performed with single-focal plane images as described in the text. Treated samples showed 63% and 55% of Waviness-% for 40 mM and 400 mM SMG, respectively (Table).

As shown in Figure 5, we also performed an analysis of images with methods adopted from the cardiovascular blood vessel literature, using a plugin module of the freely accessible “Image J” software (http://imagej.nih.gov/ij/; provided in the public domain by the National Institutes of Health, Bethesda, MD, USA), that is “Neuron J”. Regarding fiber bundle straightening in the crosslinked samples, we observed that there was greater waviness in the uncrosslinked samples using paired controls (left eye of the same animal). In other words, crosslinking resulted in straightening of fiber bundles as indicated by a decreased waviness-% (Table), as shown by the significantly lower waviness-% in 40 mM and 400 mM crosslinked sclera versus untreated control sclera. The difference in waviness between 40 and 400 mM SMG-treated samples was not statistically significant, however.

**Table:**
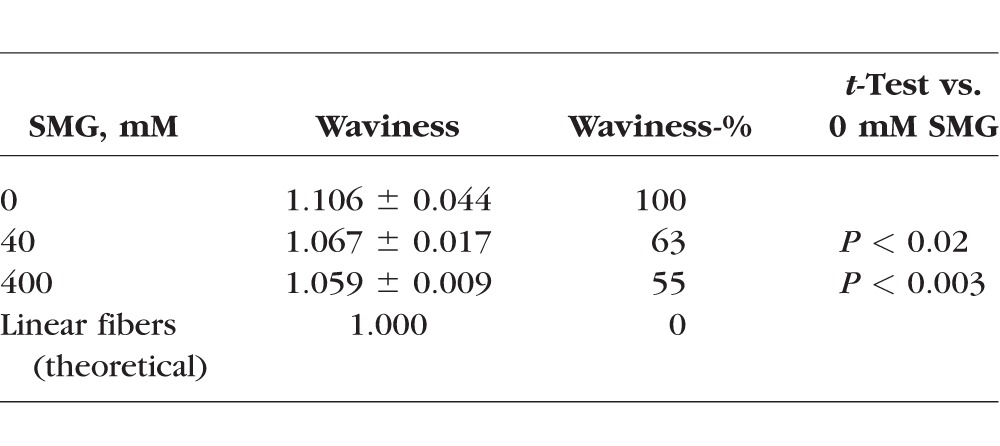
Results From Waviness Analysis of SMG-Treated Samples

## Discussion

Our experiment showed evidence supporting the use of SHG signal as a method for evaluation of collagen crosslinking effects in sclera, raising the possibility of one day using this technique as a monitoring tool for crosslinking treatments that target collagen proteins. Of note, an instrument already is in clinical use that can potentially capture this SHG signal. Although this instrument primarily was designed for imaging skin human dermis, it has been used successfully to image cornea and sclera. In our experiments, we controlled instrument “brightness” to be in a “good range” and maintained consistency throughout the experiment. Furthermore, in an effort to assure equal ionic strength of the tissue solutions, since the solution's ionic capacity can impact the SHG signal, it should be noted that the tissue was routinely placed into the same physiologic buffer (PBS) before imaging. It also should be noted that the instrument parameters that control the “gain” settings will be specific to each particular instrument, making exact comparisons between different instruments from different institutions difficult. Our findings using this technique also raise the possibility that the site of modification with chemical crosslinking, such as with SMG TXL, may be occurring along the fibril outer shell since the SHG signal has been shown to emanate from this location.

The cornea and sclera also have been evaluated concurrently in studies using this technique.^26–29^ Other studies have examined corneal tissue independently in its native state^30–36^ and in keratoconus^37,38^ as well as following photochemical crosslinking (as discussed below). The results of these studies indicate that the corneal signal is optimized in the forward scattered direction, which makes sense given the cornea's transparency and the fact that light passes through the tissue to strike a monitor in forward scattered systems. The backward scattered imaging is superior for scleral imaging, since the tissue is opaque. In this study, we took advantage of our readily available backwards scattered system studying scleral tissue collagens by their SHG signal.

With this background, it seems obvious that this imaging technique should be used to follow tissue crosslinking changes, since modification of collagens could be expected to alter the SHG signal produced. Indeed, several groups have studied the effects of the CXL technique on the SHG signal produced in the cornea.^39–43^ In a study by Steven et al.,^41^ corneal stabilization using the CXL technique resulted in a “homogenization” of signal and loss of tissue “folds” or “undulations” seen in noncrosslinked samples. These types of changes, however, also were noted in a study evaluating the effects of changes in IOP on corneal SHG signals.^41^ The “homogenization” effect in the cornea could in some way be analogous to the “straightening” that we have reported herein regarding scleral TXL with SMG. Organizationally from the fibril as well as the higher order fiber bundle/lamellar organization standpoint, the cornea and sclera are quite different and much is known about such differences from electron microscopy studies. The two tissues differ with regard to fibril packing, which includes fibril diameter distribution (small uniform fibrils for the cornea and variable diameter fibrils for the sclera) and interfibril spacing (uniform for cornea and variable for sclera). As well, the higher order organization into lamellar sheets (cornea) versus fiber bundles (sclera) is quite different. Such structural differences are reflected in the SHG signals produced by these two tissues. Thus, changes induced by crosslinking may alter the SHG signal in different but parallel ways. In other words, the “straightening” of fibers in the sclera that we observed in this study, and the “homogenization” of signal in the cornea that has been reported in the literature, are the result of collagen crosslinking.

The straightening effect may represent “fixation” of tissue while under some degree of “stretch” since the IOP did not change over the course of the experiment. In other words, this can tell us something about the degree of stabilization that is induced. The significance of this observation is unclear. In previous studies in large elastic arteries, the degree of waviness was evaluated albeit in a sophisticated 3-dimensional analysis.^44,45^ We applied a simpler image analysis technique using and Neuron J software to the images taken from our scleral crosslinked samples to quantitate potential differences in waviness between samples. The results indicate that crosslinking decreases collagen waviness in the intact globe under physiologic mechanical load (i.e., IOP). The mechanisms produced by TXL that result in this straightening effect are unclear based on the current study although one possibility is that the tissue was “fixed” in a mechanically “loaded” position and, thus, held in a “straightened” position. This would support the notion that induced “fibril stabilization” had occurred. Further studies will be necessary to determine the significance of this finding.

We also performed a regional analysis of crosslinking changes (by Tm) induced by a sT injection of SMG. As expected the level of crosslinking effect was concentrated in the area of the injection. Little or no crosslinking effect was noted in the region directly opposite (furthest away) from the injection, consistent with what is known regarding localization of effect following sT injection as shown by ultrasound localization^46,47^ and computed tomography.^48^

Finally, regarding crosslinking therapy and myopia, collagen crosslinking of the cornea is finding widespread use in the treatment of corneal destabilization including keratoconus, post-LASIK keratectasias, pellucid marginal degeneration (PMD), and as an adjunct to refractive surgical procedures.^49^ The success of treating corneal disease with crosslinking has led to the exploration of applying this treatment approach to the back of the eye and in particular, the sclera, for limiting axial elongation in high myopia,^2^ a concept that goes back to the very earliest stages of the therapeutic crosslinking concept.^50,51^
